# The Impact of Electrographic Seizures on Developing Hippocampal Dendrites Is Calcineurin Dependent

**DOI:** 10.1523/ENEURO.0014-17.2017

**Published:** 2017-04-28

**Authors:** Masataka Nishimura, J. R. Casanova, John W. Swann

**Affiliations:** 1 The Cain Foundation Laboratories, The Jan and Dan Duncan Neurological Research Institute, Texas Children’s Hospital, Houston, TX 77030; 2 Department of Pediatrics, Baylor College of Medicine, Houston, TX 77030; 3 Department of Neuroscience, Baylor College of Medicine, Houston, TX 77030

**Keywords:** development, epilepsy, fCaNB1, FK506, hippocampus, viral transfections

## Abstract

Neurobehavioral abnormalities are commonly associated with intractable childhood epilepsy. Studies from numerous labs have demonstrated cognitive and socialization deficits in rats and mice that have experienced early-life seizures. However, the cellular and molecular mechanisms underlying these effects are unknown. Previously, experiments have shown that recurrent seizures in infancy suppress the growth of hippocampal dendrites at the same time they impair learning and memory. Experiments in slice cultures have also demonstrated dendrite growth suppression. Here, we crossed calcineurin B1 (CaNB1) floxed and Thy1GFP-M mice to produce mice that were homozygous for the both the floxed CaNB1 and the Thy1GFP-M transgene. Littermates that were homozygous for wild-type CaNB1 and Thy1GFP-M served as controls. Hippocampal slice cultures from these mice were transfected with an AAV/hSyn-mCherry-Cre virus to eliminate CaNB1 from neurons. Immunohistochemical results showed that CaNB1 was eliminated from at least 90% of the transfected CA1 pyramidal cells. Moreover, the CaN-dependent nuclear translocation of the CREB transcription coactivator, CREB-regulated transcriptional coactivator 1 (CRTC1), was blocked in transfected neurons. Cell attach patch recordings combined with live multiphoton imaging demonstrated that the loss of CaNB1 did not prevent neurons from fully participating in electrographic seizure activity. Finally, dendrite reconstruction showed that the elimination of CaNB1 prevented seizure-induced decreases in both dendrite length and branch number. Results suggest that CaN plays a key role in seizure-induced dendrite growth suppression and may contribute to the neurobehavioral comorbidities of childhood epilepsy.

## Significance Statement

Seizures are thought to have a negative impact on the developing brain. This is particularly true for the epileptic encephalopathies that are commonly characterized by recurring seizures that are unresponsive to medication. Neurobehavioral abnormalities, including intellectual disabilities, are frequent in these patient populations. Research in numerous animal models has been able to reproduce many aspects of these clinical syndromes including learning and memory deficits. However, the cellular and molecular mechanisms contributing to cognitive decline are only now beginning to be revealed. In *in vitro* experiments reported here, we extend our studies of seizure-induced abnormalities in developing hippocampal dendrites and show that the calcium calmodulin dependent protein phosphatase, calcineurin (CaN) plays a key role in electrographic seizure-induced reductions in dendrite length and branching complexity.

## Introduction

Calcineurin (CaN) is a calcium calmodulin dependent serine/threonine protein phosphatase that is expressed at high levels in the mammalian central nervous system (Klee et al., 1998; Baumgärtel and Mansuy, 2012). It has been shown to have important roles in how neurons respond to alterations in network activity and concomitant changes in intracellular calcium concentrations. CaN is a heterodimer, composed of a catalytic subunit, CaNA and a regulatory subunit CaNB (Klee et al., 1998). While three genes encode the α, β, and γ isozymes of CaNA, two genes encode the CaNB1 and CaNB2 isoforms. CaNB2 is not present in the brain, but CaNB1 is there in abundance (Ueki et al., 1992; Chang et al., 1994).

Attempts to eliminate CaNAα from the brain to study CaN’s roles in synaptic transmission and plasticity have proved difficult (Zhuo et al., 1999) since CaN activity is only partially reduced, due to the presence of CaNAβ (Zeng et al., 2001). In subsequent experiments, CaNB1 was selectively eliminated from forebrain glutamatergic neurons in a CaNB1 “floxed” line of mice (Zeng et al., 2001). This resulted in a loss of both CaNB1 and CaNA. The later reduction was suggested to be due to the instability of CaNA when its regulatory subunit CaNB1 was absent.

When synaptic plasticity was studied in hippocampal slices from CaNB1 knock-out mice, LTD was impaired in CA1 pyramidal cells (Zeng et al., 2001). These results were in good agreement with experiments that showed that CaN inhibitors suppress LTD induction (Kirkwood and Bear, 1994; Mulkey et al., 1994; Hodgkiss and Kelly, 1995; Malenka and Bear, 2004). Several mechanisms have been suggested to explain CaN’s role in LTD (Beattie et al., 2000; Lee et al., 2000; Lin et al., 2000). One is that CaN mediates a collapse of dendritic spines and the activity-dependent elimination of glutamatergic synapses (Zhou et al., 2004).

CaN has been shown to act over both short and extended time periods in modifying neuronal function. Studies of LTD are an example of CaN mediating rapid responses. CaN is well known to not only dephosphorylate GluR1(Man et al., 2007); the synaptic protein involved in LTD but also A-type potassium channels (Lin et al., 2011), the metabotropic glutamate receptor mGluR5 (Alagarsamy et al., 2005) and GABAa receptor subunits (Jones and Westbrook, 1997). Moreover, CaN is known to target the cytoskeletal protein actin via cofilin a key regulator of actin filament dynamics (Wang et al., 2005). In so doing, it mediates rapid morphologic alterations in dendritic spines and axon growth cones. Over extended time periods, CaN is well known to dephosphorylate cytoplasmic subunits of the Nuclear Factor of Activated T cells (NFAT) transcription complex, which leads to its nuclear import and alterations in gene transcription (Flanagan et al., 1991; Crabtree and Olson, 2002). More recently, it has been shown to similarly activate the transcription cofactor CREB-regulated transcriptional coactivator 1 (CRTC1) and translocate it to the nucleus (Ch'ng et al., 2012).

Comparative few studies have examined the role of CaN in the development of the nervous system (Graef et al., 2003). However, its presence throughout embryonic development is essential for survival (Graef et al., 2001). Later during early postnatal development, CaN appears to have a role in fine tuning neurite growth, particularly dendrite growth by limiting growth rates (Okazawa et al., 2009; Schwartz et al., 2009).

Our laboratory has been interested in the mechanisms underlying learning deficits following recurring seizures in early life (Casanova et al., 2012). Our previous results showed that seizures suppress the growth of dendrites (Nishimura et al., 2011). A major effort is now underway to identify the molecular events underlying growth suppression. Since seizures are accompanied by a massive increase in intracellular calcium, we have pursued a role for calcium signaling mechanisms in the impact seizures have on developing dendrites. Along these lines, we previously reported that recurring seizure-like activity in slice culture preparations results in a surprisingly rapid reduction in CA1 hippocampal pyramidal cell dendrite length. This occurred within 4 h and was blocked by CaN inhibitors (Casanova et al., 2013). Here, we extend these observations by inducing electrographic seizures for much longer periods of time (2 d) and use genetic strategies to eliminate CaNB1 from CA1 hippocampal pyramidal cells. CaNB1 floxed mice and Thy1GFP-M mice were crossed which enabled a detailed analysis of dendritic arbors. In slice cultures, we eliminated CaNB1 and functional CaN activity from CA1 hippocampal neurons by transfections with an AAV-Cre virus and induced seizure-like activity with bicuculline. Our results show that CaN plays a key role in electrographic seizure-induced dendrite growth suppression.

## Materials and Methods

### Animals

CaNB1 floxed mice also referred to as the fCaNB1 line (Zeng et al., 2001) were purchased form The Jackson Laboratory. They were crossed with the Thy1GFP-M mouse line (Feng et al., 2000) to produce a mouse line that was homozygous for the CaNB1 floxed gene as well as the Thy1GFP-M transgene (fCaNB1/fCaNB1, Thy1GFP-M/Thy1GFP-M). These mice were crossed with mice that were homozygous for Thy1GFP-M to produce offspring that were heterozygous for the CaNB1 floxed gene and homozygous for the GFP transgene [fCaNB1/wildtype (WT), Thy1GFP-M/Thy1GFP-M]. F1 littermates from fCaNB1/WT, Thy1GFP-M/Thy1GFP-M male and female pairs were genotyped by genomic PCR. Mice that were homozygous for fCaNB1 (fCaNB1/fCaNB1, Thy1GFP-M/Thy1GFP-M) and wild-type CaNB1 (WT/WT, Thy1GFP-M/Thy1GFP-M) were used in the experiments reported here. Since all mice were homozygous for the Thy1GFP-M gene and expressed GFP in CA1 hippocampal pyramidal cells, this facilitated the examination of alterations in dendrite microanatomy. All mice were on a C57BL6 background. No attempt was made to select the sex of animals for study thus male and female mice were randomly assigned to groups in all experiments reported here.

There was one series of experiments in which we did not use fCaNB1 mice. These were studies of the time course of activity-dependent CRTC1 nuclear translocation. For these experiments, C57BL6 mice were used. The maintenance of animals and surgical procedures were approved by the Baylor College of Medicine institutional animal care committee and were in keeping with guidelines established by the National Institutes of Health.

#### Hippocampal culture preparation

Slice cultures of the hippocampus were made as previously described (Stoppini et al., 1991; Nishimura et al., 2008). Briefly, the day of birth was designated as postnatal day 0 and transverse hippocampal slice cultures were prepared from 5-d-old control CaNB1wild-type mice (WT/WT, Thy1GFP-M/Thy1GFP-M) and from littermate fCaNB1 mice (fCaNB1/fCaNB1, Thy1GFP-M/Thy1GFP-M). Animals were deeply anesthetized with isoflurane vapors, and the forebrain was removed and submerged in cooled Leibovitz’s L-15 culture media (Invitrogen). The hippocampi were dissected free of adhering tissue and placed on a Teflon stage of a custom-built mechanical tissue chopper, with a small amount of L-15 media. Transverse (375 µm) slices were cut and placed on warmed Millicell membrane inserts (Millipore) presoaked in normal culture media consisting of 98% Neurobasal A (Invitrogen), 2% B27 (Invitrogen), and 0.5 mM glutamine (Sigma). Three slices were put on each insert and arranged such that they did not contact one another. The Millicell membranes were then placed in six-well plates and placed in a water-jacketed incubator at 37°C with 5% CO_2_ atmosphere. The culture media were changed at 2-d intervals. Following 3 or 4 d *in vitro* (DIV), randomly selected slices were treated with 100 µM bicuculline methobromide (Sigma).

### Viral transfection

The adeno-associated virus, rAAV8/hSyn-mCherry-Cre (AAV-mCherry-Cre), was purchased from The University of North Carolina Gene Therapy Center vector Core. To transfect slice cultures 1 µl of the viral solution (titer ranged from 3 to 6 × 10^12^/ml) was applied directly to the slice surface 1-2 h after slice preparation.

### Immunohistochemistry of CaNB1

At the completion of experiments, slice cultures were fixed with 4% paraformaldehyde/4% sucrose. Slices were carefully lifted from the Millicell membranes, placed into individual vials, and rinsed free-floating in 1× PBS. For the following protocol, all immunohistochemical reactions in experimental and control slice cultures were done simultaneously under identical conditions. First, slices were rinsed twice in PBS for 10 min each and then once in PBS with 0.3% Triton X-100 (Sigma) for 1 h. The slices were then incubated in a solution containing the primary antibodies for 24 h at 4°C. This solution consisted of 1× PBS, 0.3% Triton X-100, the primary rabbit antibody, anti-rabbit CaNB antibody (1:1000, #07-069, Millipore), and the Mouse NeuN antibody (1:1000, MAB377, Millipore). The next day, the slices were rinsed three times in PBS and then incubated for 2 h with Alexa Fluor 647-conjugated goat anti-rabbit secondary antibody (1:500, Invitrogen) and Alexa Fluor 405-conjugated goat anti-mouse secondary antibody (1:500, Invitrogen) dissolved in PBS. Afterward, the tissue was again rinsed three times in PBS and all sections were then mounted on slides with Fluorescent Mounting Medium (Dako) and glass coversliped. Images of Alexa Fluor 405 and Alexa Fluor 647-labeled neurons were obtained using a Diode 405-430 laser (405-nm line) and He/Ne laser (633-nm line) of a Zeiss710 confocal microscope respectively. We used Zen2010 software for image acquisition (Zeiss). Overlaid (merged) images were produced by sequential excitation of Alexa Fluor 405, mCherry, and Alexa Fluor 647. In some instances, pseudo-coloring was used for image clarity.

To quantify bicuculline-induced alterations in the CaNB–Alexa Fluor 647 signal in slice cultures, confocal images (single optical section, 135 × 135µm) were obtained with a Plan-Apochromat 63× Oil DIC M27 objective [numerical aperture (NA) 1.40] and were analyzed by using ImageJ software (NIH). For all CA1 neurons that demonstrated mCherry signals in their nuclei, the number of neurons which had nondetectable or detectable levels of CaNB1 in cell bodies were counted in a blinded fashion. Two randomly-selected areas from the CA1 region of every slice were analyzed and averaged. A total of 18 slice cultures (10 from CaNB1 WT mice and 8 from fCaNB1 mice at DIV4) were analyzed.

#### Analysis of CRTC1 translocation

To determine the time course of CRTC1 (also known as TORC1) translocation induced by bicuculline treatment, hippocampal slice cultures were prepared from C57BL6 mice and treated with 100 µM bicuculline on DIV3 for 15 min to 8 h. Cultures were fixed immediately following treatments and slices were double immunostained with rabbit-CRTC1 monoclonal antibody (1:1000, #2587, Torc1 [C71D11], Cell Signaling Technology) and mouse-NeuN (1:1000) antibody. Alexa Fluor 488-rabbit and Alexa Fluor 647-mouse antibodies were used as secondary antibodies. Nuclei were stained with DAPI. Using a 63× oil objective on a Zeiss 710 confocal microscope, two images from randomly-selected regions of area CA1 were taken from each of three slices (per experimental condition, total six images). Images were then analyzed using ImageJ software. In each image, one investigator randomly selected six neurons in a blinded fashion from images of NeuN staining and another investigator used ImageJ software to measure the intensity of CRTC1 immunofluorescence in the nucleus of these cells, which was identified by DAPI staining. We also measured the intensity of the CRTC1 signal in the surrounding perikaryal cytoplasm of the same cells which was identified by NeuN staining. We excluded all DAPI-stained nuclear regions from our measures of cytoplasmic CRTC1. We used the very low CRTC1 signals in the small nuclei of nearby glial cells (confirmed by GFAP immunohistochemistry) in each image as background intensity. Nucleus/cytoplasm (N/C) ratios of CRTC1 were then calculated. The N/C ratio of the two images from each slice was averaged. Overlaid (merged) images were produced by sequential excitation of Alexa Fluor 488 and Alexa Fluor 647. Experiments were repeated 3 times. Thus, our database consisted of N/C ratios from nine slices (three slices × three experiments) per experimental condition.

To validate CaNB1 knock-out by virus transfection, we used activity-dependent CRTC1 translocation as a biomarker for CaN activity. In these experiments, we studied CRTC1 translocation after a 4-h bicuculline treatment. Hippocampal slice cultures were prepared from CaNB1 (control) and fCaNB1 (experimental), Thy1GFP-M mice and transfected with the AAV-mCherry-Cre virus 1-2 h later. Half of the slices from control and experimental mice were treated with 100 µM bicuculline for 4 h on DIV4. Following fixation, slices were immunostained with rabbit-CRTC1 antibody and Alexa Fluor 647-rabbit antibody was used as the secondary antibody. Analysis of CRTC1 translocation were conducted using the same techniques described above in the analysis of the time course of bicuculline-induced CRTC1 translocation with the exception that mCherry was used as a way to identify transfected cells and demarcating nuclear regions of the soma of neurons. As before, images from three slices from three experiments for every experimental condition were analyzed. Overlaid (merged) images were produced by sequential excitation of mCherry and Alexa Fluor 647.

#### Neuron tracing and analysis of dendrite anatomy

Slice cultures were prepared from CaNB1, Thy1GFP-M (control) and fCaNB1, Thy1GFP-M (experimental) mice and transfected on DIV0 with the AAV-mCherry-Cre virus. At the completion of experiments, slices were fixed overnight in 4% paraformaldehyde/4% sucrose buffered saline at 4°C. Thereafter, they were washed with 1× PBS and mounted on slides. CA1 neurons that had strong GFP fluorescence throughout their dendritic trees and were well isolated from other GFP neurons and also showed mCherry in their nuclei were randomly selected by an investigator blinded to experimental conditions and imaged with a Zeiss 710 confocal microscope at 1024 × 1024 pixel resolution for morphologic analysis. Basilar dendritic arbors were reconstructed digitally from the image stacks using NEUROLUCIDA software (MicroBrightField). It was not possible to reconstruct CA1 apical dendrites from GFP-M mice due to the large number of fluorescent processes in the apical dendritic layer. GFP images were acquired via excitation with an Argon laser (488 nm) and ECPlan-Neofluar 40× Oil DIC M27 objective (NA 1.3; Zeiss) with 0.6 zoom using the appropriate manufacturer-suggested confocal apertures. Steps in the *z*-axis were in 2-µm increments and Kalman accumulation averaging of 2 was used. All Neurolucida reconstructions were conducted in a blinded manner. Quantitative analysis of the traced data were performed using NEUROEXPLORER software (MicroBrightField). Numerical data (total dendritic length, number of branch points and number of intersections by Sholl analysis), such as geometric means and SEMs, were then calculated for each experimental group. Each experiment was repeated on three separate occasions and results combined for the final analysis.

#### Multiphoton microscopy and electrophysiology

Slice cultures were prepared as described above, and following 3-7 DIV in normal defined media, the Millicell membrane inserts were submerged in a custom machined imaging/recording chamber and allowed to equilibrate for 15 min in warmed (30°C), oxygenated (95% O_2_, 5% CO_2_) artificial CSF (ACSF). ACSF was constantly perfused throughout the duration of the experiment and contained: 1.5 mM CaCl_2_, 1.5 mM MgSO_4_, 122.75 mM NaCl, 3.5 mM KCl, 10 mM glucose, 26 mM NaHCO_3_, and 1.25 mM NaH_2_PO_4_. A region of interest in CA1 containing Thy1-GFP-positive neurons was identified through brief excitation with a X-Cite 120Q mercury vapor short arc lamp (Lumen Dynamics) and visualized by a Zeiss LSM 7 MP microscope using a 20× water Plan-Apochromat objective (NA 1.0) or Nikon 40× water Plan-Apochromat objective (NA 0.8) equipped with a Zeiss GFP bandpass filter (500-550 nm). To visualize CRE-transfected neurons, GFP and mCherry were coexcited at 760 nm using a Chameleon Ultra II Ti:Shapphire laser where GFP and, Cherry fluorophore emissions were simultaneously detected by a Zeiss BiG photodetector internally equipped with Zeiss Filterset 2 (BP 500-550 nm and BP 565-610 nm). Once a potential transfected neuron was identified, a three dimensional volume (212 × 212 × 100 µm, 1-µm step) was acquired to confirm the nuclear localization of mCherry within the pyramidal neuron of interest. Before tuning the laser wavelength to 920 nm and performing electrophysiological studies, the depth (*z*-position) of the neuronal soma was stored within the hardware interface of the Zeiss motorized axis controller and its outline was overlaid on screen with a drawing marker available in Zeiss’ ZEN2011 acquisition software.

A Sutter Instruments P-1000 puller was used to fabricate borosiliate glass (Sutter) microelectrodes with 3- to 6-MΩ resistance. The electrodes were filled with Alexa Fluor 488 hydrazide/ACSF. While scanning live at 920 nm, PatchStar micromanipulators (Scientifica) controlled by LinLab 2 (Scientifica) were used to lower an electrode into the stratum pyramidale adjacent to the neuron of interest to record local field potentials while, a second recording electrode was positioned to obtain a loose cell-attached recording. During recordings, 2-photon live scanning was terminated. Patches were voltage clamped at 0 mV using a Molecular Devices Multiclamp 700B amplifier and raw signals were filtered at 4 kHz before being digitized by an Axon 1440 digitizer set to sample at >10 kHz. Following 15 min of baseline recordings in ACSF, the perfusion solution was switched to ACSF media containing 50 µM bicuculline methiodide. The resulting spontaneous epileptiform activity of the local field potentials in addition to action potentials from the target neuron were recorded for an additional 15 min. At the termination of the experiment a final membrane test was preformed to ensure the seal resistance did not vary >10% of the initial starting values and that rupture of the membrane and entrance into the neuron had not occurred. We used a higher concentration of bicuculline (100 µM) in our anatomic studies to avoid a potential loss of drug effectiveness due to the prolonged incubation times (up to 48 h) at the higher incubator temperatures.

### Cross-correlation data analysis

A custom MATLAB graphics user interface was developed to correlate bicuculline-induced spontaneous spike timing of the CA1 population with action potentials of individual transfected CA1 pyramidal cells. We analyzed recurring periods of synchronized network bursting and analyzed recordings 2000 ms before and after the onset of each network discharge. Briefly, abf2load.m (mathworks file ID #22114) was used to import pClamp data files and the timestamp of population spikes in local field potential recordings and action potentials in a neuron were detected using peakfinder.m, (file ID #25500). These time stamps were then used to calculate the difference in the time of population spiking and spiking in individual neuron. The resulting database for each experiment consisted of differences in spike timing between the population and transfected neuron. Results from 4 experiments were compiled, averaged, binned at 1-ms intervals and plotted as cross-correlgrams. For comparison, the time stamps for action potentials in the pyramidal cell in each experiment were randomly reassigned a new time during each recording epoch. Cross-correlations were then computed to represent these spurious correlations. Statistics were calculated based on comparisons between the original and randomized datasets.

### Statistics

ORIGINPRO version 9.1 (OriginLab) was employed for statistical analysis and for the construction of histograms and graphs. Data were analyzed statistically using a one- or two-way ANOVA followed by a Fisher’s least significant difference test to correct for multiple comparisons. For the analysis of cross-correlations in electrophysiological recordings, data were found not to be normally distributed using the Shapiro-Wilk normality test. Therefore, a Kruskal-Wallis ANOVA was employed followed by a Mann–Whitney *U* nonparametric test. All data are presented as the mean ± SEM.

## Results

Previous studies have shown that CaN is widely expressed in the nervous system and throughout the cytoplasm of the soma, dendrites, and axons but absent from nuclei of neurons (Kuno et al., 1992). In the hippocampus, it is present in all hippocampal laminae and prominent in the dendritic layers of area CA1 and CA3 (Zeng et al., 2001). CaN is also expressed during brain development (Eastwood et al., 2005), and the pattern of CaN immunostaining we have observed in slice cultures prepared from infants mice is very reminiscent of previous reports from adulthood. CaNB1 is highly expressed throughout area CA1 both in the stratum oriens and radiatum dendritic layers as well as in stratum pyramidale.

### Impact of AAV-Cre viral transfections on the expression of CaNB1

**Figure 1.**
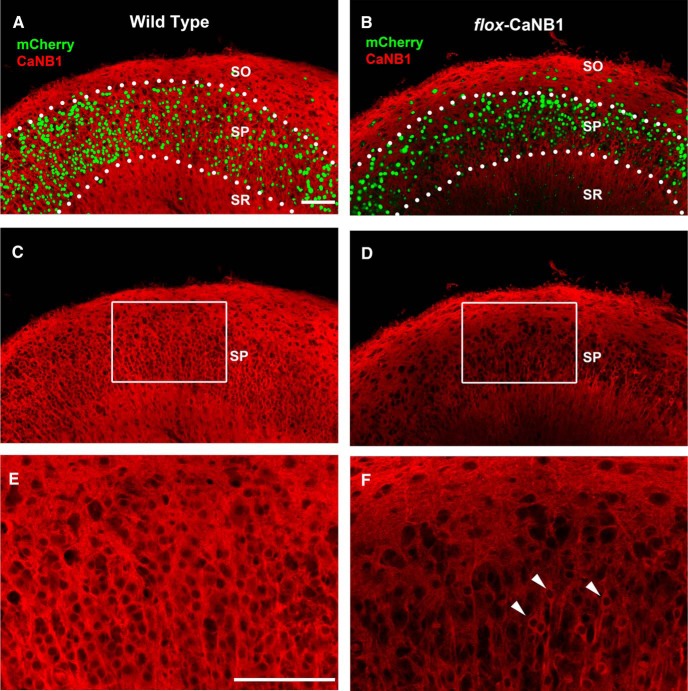
Transfection with rAAV8/hSyn-mCherry-Cre in an fCaNB1 mouse slice cultures results in a reduction in CaNB expression. ***A***, ***B***, Low-magnification confocal images of area CA1 showing the transfection of a large number of neurons. mCherry (shown here as green) which is fused to Cre serves as a reporter of viral transfection. ***C***, Image illustrates the typical widespread distribution of CaNB in area CA1 from wild-type mice. The protein is highly expressed in basal (stratum oriens, SO) and apical (stratum radiatum, SR) dendritic layers. ***E***, Area in ***C*** outlined by box and shown at higher magnification. Notable is the lack of immunoreactivity in cell nuclei, particularly in the cell bodies of stratum pyramidale (SP). ***D***, AAV-Cre transfection of fCaNB1 mouse cultures resulted in a marked reduction in CaNB1 immunoreactivity. ***F***, Area in ***D*** outlined by box and at higher magnification. Intermixed with transfected cells are untransfected neurons (see arrowheads) in which the cytoplasm and somas and apical dendrites stain intensely for CaNB1. The staining of these untransfected neurons stand out against a background of immunonegative transfected neurons. Tissue was processed on DIV8. Scale bars, 100 µm (***A–D***) and 300 µm (***E***, ***F***).

Under our experimental conditions, when the AAV-mCherry-Cre was applied to slice cultures many neurons were transfected. This is illustrated by the widespread expression of nuclear mCherry in Figure 1*A*,*B*, where the mCherry is pseudo-colored green. The AAV we used carried a gene for a mCherry-Cre fusion protein that was under the control of the synapsin promoter. This permitted expression of Cre only in neurons and the visualization of transfected cells by mCherry in nuclei (due to its fusion with Cre). When AAV-mCherry-Cre was applied to slice cultures from wild-type mice, we observed no noticeable changes in the CaNB1 immunostaining. Robust immunoreactivity was observed in the dendritic laminae as well as in the cell body layer (Fig. 1*A*,*C*,*E*). And just as reported previously, the nuclei of cells were nearly devoid of immunoreactivity (Fig. 1*E*). In contrast, when slices from fCaNB1 mice were transfected, there was a dramatic decrease in CaNB1 levels in area CA1 (Fig. 1*B*,*D*,*F*), compared with transfected slices from wild-type animals. However, not every cell was transfected and those that were not had markedly higher levels of CaNB1 than nearby transected neurons. Arrow heads in Figure 1*F* denote untransfected cells. Notable are the high levels of CaNB1 in their perikaryal cytoplasm as well as in apical dendrites as they project through stratum pyramidale into stratum radiatum. Also noticeable is the relative low levels of CaNB1 in the nuclei of these untransfected neurons, which is in keeping with images in Figure 1*C*,*E*.

**Figure 2.**
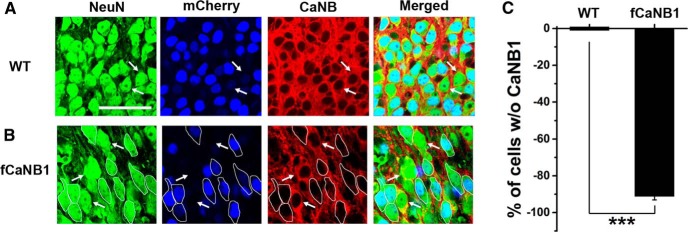
AAV/hSyn-mCherry-Cre transfections result in a marked reduction in CaNB1 expression in transfected neurons from fCaNB1 mice but had no effect of CaNB1 expression in neurons from wild-type (WT) mice. NeuN immunoreactivity was used to stain neuronal cytoplasm and nuclei. Nuclear mCherry reports viral transfection and CaNB1 immunoreactivity is shown in red. ***A***, Transfected neurons in slices from wild-type mice showed no discernable effect on CaNB1 expression. Arrows point out two untranfected cells. ***B***, In contrast, viral transfections in slices from fCaNB1 mice resulted in a complete loss of CaNB1 immunoreactivity which is markedly different from the immunoreactivity of untransfected cells (arrows). ***C***, Quantification of results from 10 wild-type and 8 fCaNB1 mouse slices showed that over 90% of transfected cells from fCaNB1 mice had no detectable CaNB1 in their cytoplasm but virtually every transfected neuron from wild-type cells had high levels of CaNB1. Scale bar, 50 µm; ***α = 0.001.

To quantify the ability of AAV-mCherry-Cre transfections to knock-out CaNB1 in neurons from fCaNB1 mice, we doubled labeled CA1 neurons with the neuronal marker NeuN and CaNB1 and relied on mCherry to identify neurons that were transfected. Images from both wild-type and fCaNB1 littermate mouse cultures are shown in Figure 2. Wild-type mouse cultures (Fig. 2*A*) show a uniform cytoplasmic colabeling of neurons for NeuN and CaNB1 in both transfected (mCherry-positive) and untransfected neurons. This is seen most clearly in the merged image where the cytoplasm of neurons is yellow. In contrast, neurons from slices from fCaNB1 mice fell into two distinct populations. Neurons that were transfected (some of which are outlined in Figure 2*B*) were essentially devoid of CaNB1 protein but easily visualized by NeuN while untransfected cells had high levels of both NeuN and CaNB1 (Fig. 2*B*, arrows). In the merged image in Figure 2*B*, the cytoplasm of transfected neurons appears green, denoting the presence of NeuN and the absence of CaNB1, while the cytoplasm of untransfected cells is yellow indicating the presence of both proteins. To quantify these results, we counted the number of transfected neurons (*N* = 928 neurons total) in slice cultures from both wild-type and fCaNB1 mice that showed no detectable cytoplasmic CaNB1 immunoreactivity in their cell bodies. Nearby untransfected neurons served as an internal reference in all slices examined. Images from 18 slice cultures were examined, of which 497 transfected neurons were counted across 10 slices from wild-type mice and 431 transfected neurons from 8 slices from fCaNB1 mice. Cumulative results in Figure 2*C* show that 91.20 ± 1.91% (*p* = 4.57 × 10^−10^, *t*_(7)_ = 47.83) of transfected neurons from fCaNB1 mice had little if any CaNB1 immunoreactivity but every neuron from wild-type mice had high, and apparently, unaltered levels of this protein.

### Elimination of CaNB1 prevents activity-dependent nuclear translocation of CRTC1

While our immunohistochemical results indicated that transfections with the AAV-mCherry-Cre virus appeared to be quite effective in eliminating CaNB1 from CA1 hippocampal pyramidal cells, we sought an additional way to verify that CaN activity was eliminated from transfected neurons. CRTC1 has been shown to play important roles in activity-dependent synaptic plasticity in the central nervous system (Zhou et al., 2006; Kovacs et al., 2007). Under baseline conditions, CRTC1 is cytoplasmic and distributed not only in the soma of hippocampal pyramidal cells but throughout their dendrites (Ch'ng et al., 2012). However, the influx of calcium that accompanies synaptic activity has been shown to lead to a very rapid translocation of CRTC1 to the nucleus (Ch'ng et al., 2012). Early studies in pancreatic β islet cells showed that CRTC1 is phosphorylated in the cytoplasm and a CaN-dependent dephosphorylation of CRTC1 leads to its dramatic nuclear translocation (Conkright et al., 2003; Iourgenko et al., 2003). CaN has been shown to play an identical role in activity-dependent movement of CRTC1 in CA1 hippocampal pyramidal cells (Ch'ng et al., 2012). Thus, we reasoned that if CaN activity was truly eliminated from transfected CA1 pyramidal cells in our slice cultures, then neuronal activity that normally shuttles CRTC1 to nuclei should be ineffective and CRTC1 should remain in the cytoplasm in transfected cells.

**Figure 3.**
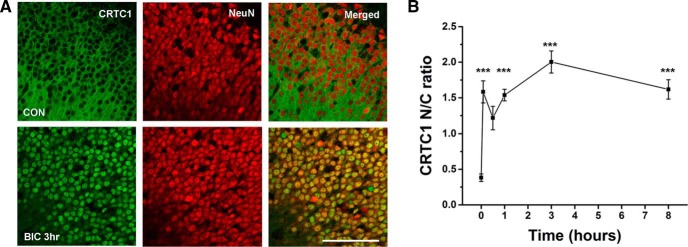
Acute treatment with the bicuculline results in a dramatic nuclear translocation of the CREB transcriptional coactivator CRTC1. ***A***, Under baseline conditions, CRTC1 is cytoplasmic and largely excluded from neuronal nuclei which are immunostained for NeuN. However, 3 h after initiating treatment with bicuculline (BIC 100 µM), CRTC1 has shuttled to the nuclei of virtually every neuron in this field of view. ***B***, The rapid time course of CRTC1 nuclear translocation. Here, the ratio of the intensity of nuclear to cytoplasmic CRTC1 immunostaining is computed at 0 min, 5 min, 30 min, 1 h, 3 h, and 8 h of bicuculline treatment. The graph summarizes results from three separate experiments, three slices per experiment, thus, *n* = 9 for each of the six time points. Scale bar, 100 µm; ***α = 0.001.

To confirm CaNB1 knock-out in transfected cells, we first examined whether bicuculline-induced epileptiform activity in wild-type slice cultures could induce CRTC1 translocation to nuclei. The images of the CA1 cell body layer in Figure 3*A* show that at baseline CRTC1 is excluded from nuclei (see CON, top row). Here, neuronal nuclei and cytoplasm are stained by NeuN and the merged images show that CRTC1 does not colocalize with nuclear NeuN. However, after 3 h of bicuculline treatment (100 µM), CRTC1 was dramatically translocated to nuclei. The colocalization of CRTC1 and NeuN is clearly evident by the yellow color in the merged image. Figure 3*B* summarizes our analysis of the time course of these effects from nine separate slices treated in three separate experiments. Here, we plot the ratio of the intensity of nuclear CRTC1 staining to cytoplasmic staining. The results demonstrate a significant and very rapid 5-fold increase in the nuclear to cytoplasmic staining ratio within 3 h of initiating bicuculline treatment (2.00 ± 0.14), compared with baseline (0.38 ± 0.05; *p =* 1.59 × 10^−11^, *t*_(16)_ = 8.76).

**Figure 4.**
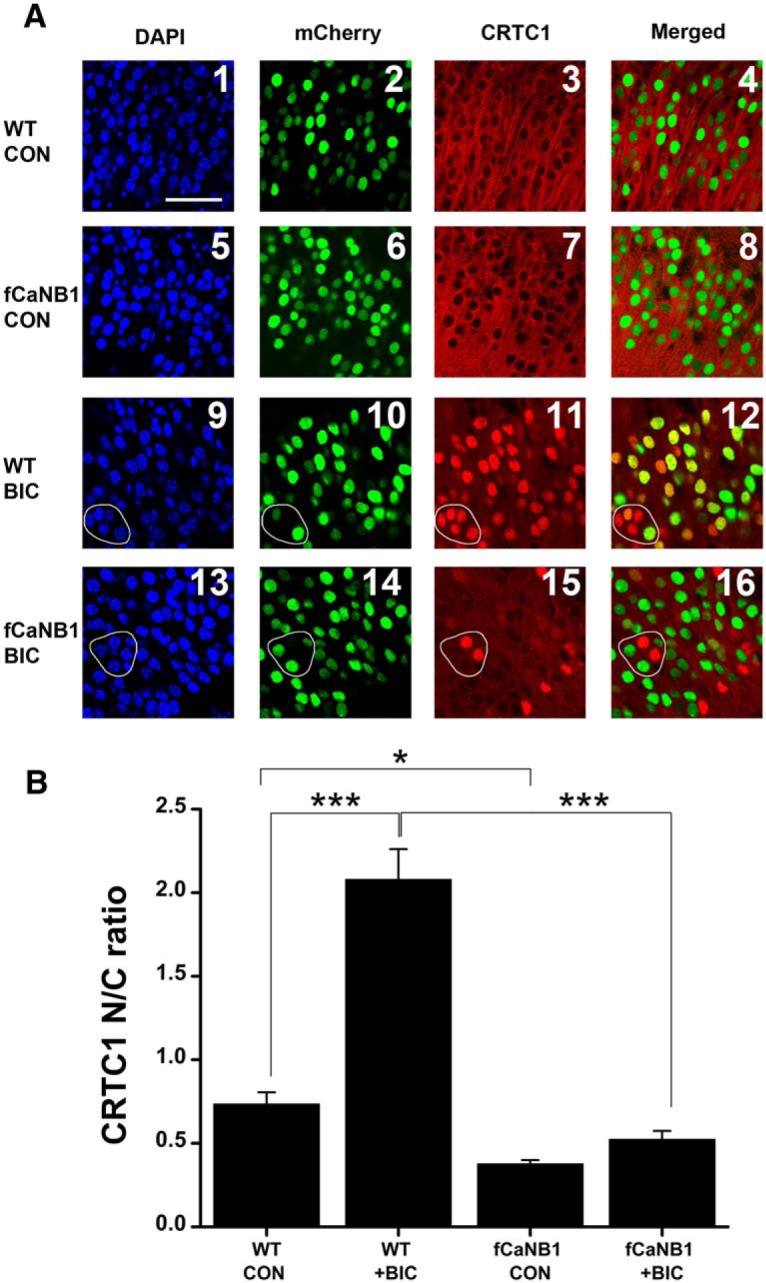
mCherry-Cre viral transfections of neurons in fCaNB1 mouse slices prevents the nuclear translocation of CRTC1. DAPI is used to stain nuclei while mCherry (shown here in green) reports neuronal transfections. ***A1–A8***, Under control (CON) conditions in both wild-type (WT) and fCaNB1 slices, CRTC1 is cytoplasmic and excluded from nuclei. ***A9–A12***, In slices from wild-type mice, bicuculline (BIC) treatment (100 µM for 4 h) resulted in CRTC1 nuclear translocation whether the neuron was transfected or not. To illustrate, five cells are circled in ***A9***. Two of which were transfected (***A10***) and CRTC1 was translocated to the nuclei of all five cells (***A11***, ***A12***). ***A13–A16***, In contrast are results from fCaNB1 mice, where bicuculline treatment did not induce a CRTC1 nuclear translocation in transfected cells but did so in untransfected cells. Five representative cells are circled in ***A13***. Three of these were transfected (***A14***). Bicuculline treatment resulted in the nuclear translocation of CRTC1 in the two untransfected cells but not in the three transfected neurons (***A15*** and ***A16***). ***B***, The computed ratio of the intensity of nuclear to cytoplasmic CRTC1 immunostaining was significantly increased in transfected neurons from wild-type mice but was not altered in transfected cells from fCaNB1 mice. The bar graphs summarize results from three separate experiments, nine slices total for each of the four conditions. Scale bar, 50 µm; *α = 0.05; ***α = 0.001.

Next, we compared bicuculline-induced CRTC1 translocation in slice cultures from wild-type and fCaNB1 mice. We combined DAPI as a nuclear marker and mCherry to report neuron transfections with CRTC1 immunohistochemistry. Under control conditions in slices from both wild-type and fCaNB1 mice, CRTC1 was excluded from neuronal nuclei in both transfected and untransfected cells. In keeping with results from Figure 3, a 4-h bicuculline treatment (100 µM) of slices from wild-type mice resulted in a dramatic translocation of CRTC1 to nuclei. This was true whether a neuron had or had not been transfected. For instance, in the group of five neurons circled in Figure 4*A9* , two cells were transfected and three were not in Figure 4*A10*. However, CRTC1 was shuttled to the nucleus in all five cells during bicuculline treatment (Fig. 4*A11*, *A12*). In contrast, when slices from fCaNB1 mice were treated with bicuculline, CRTC1 did not translocate to the nuclei of neurons that were transfected but did in nontransfected neurons. For instance, five neurons are circled in the image in Figure 4*A13*, three of the cells were transfected (Fig. 4*A14*, mCherry) and none of them displayed nuclear staining for CRTC1 (Fig. 4*A14*,*A15*). However, the two untransfected cells had robust nuclear CRTC1 staining. We quantified these results as we had done before by computing the ratio of the nuclear to cytoplasmic staining intensity for CRTC1 in transfected neurons under the four experimental conditions. To do this we conducted three separate experiments and averaged the nuclear-to-cytoplasmic ratio in 12 cells from three slices in each experiment. Thus, for each of the four experimental conditions data from a total of nine slices and 108 CA1 pyramidal cells formed the basis for our analysis. Similar to results in Figure 3*B*, our analysis showed that bicuculline treatment in slice cultures from wild-type mice resulted in a 3-fold increase in the nuclear-to-cytoplasmic ratio (Fig. 4*B*; 2.07 ± 0.07, *p =* 9.45 × 10^−11^, *t*_(16)_ = 9.43). However, in bicuculline treated slices from fCaNB1 mice, there was no increase in this ratio in transfected neurons (0.52 ± 0.50) compared with untreated fCaNB1 or wild-type slices (0.38 ± 0.02 and 0.73 ± 0.07), respectively. Clearly suggesting that CaN is no longer functional in transfected neurons following bicuculline treatment (+bicuculline; WT vs fCaNB: *p =* 2.64 × 10^−12^, *t*_(16)_ = 10.90). Results also indicate that under control conditions, elimination of CaNB1 reduced the nuclear-to-cytoplasmic ratio, indicating a possible role for CaN is shuttling CRTC1 to nuclei under the baseline physiologic conditions (*p =* 0.016, *t*_(16)_ = 2.50).

### Elimination of CaNB1 from hippocampal pyramidal cells does not impair their participation in synchronized network activity

**Figure 5.**
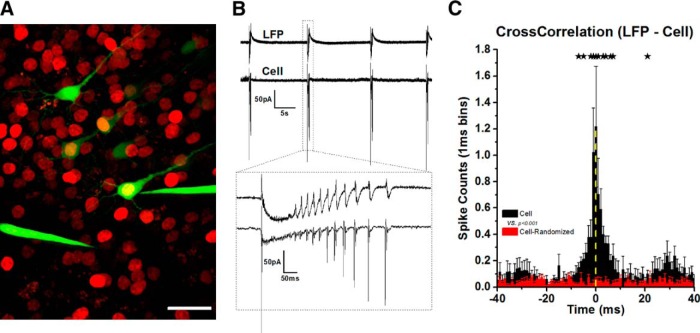
Elimination of CaNB from hippocampal pyramidal cells does not impair their participation in seizure activity. ***A***, Multiphoton image obtained during a physiology recording session. ***B***, Simultaneous local field potential (LFP) recordings and cell attached patch recordings (Cell) of seizure-like activity induced by bicuculline (50 µM). The cellular recordings were obtained from the centermost GFP-positive pyramidal cell in ***A***. This cell lacks CaNB, since it has been virally transduced and expresses mCherry in its nucleus (seen here as yellow). Recordings suggest the spiking in the cell occurs nearly simultaneous with the synchronous discharging of the population. ***C***, This correlation is verified by a cross-correlation analysis of population spikes with spikes in individual pyramidal cells in four separate experiments. Yellow dashed line indicates when population spikes occurred. The large peak centered on 0 ms shows that the pyramidal cells usually fired within a few milliseconds before or after the discharge of the population. When the time stamp for every cell action potential was randomly assigned a time during the field potential recordings no correlation was observed (red overlay), and this differed significantly from the original data. Stars indicate significant (*p* ≤ 0.05) differences between original and randomized datasets at the indicated time points. Scale bar, 25 µm.

Given the importance of CaN in neuronal physiology, it seemed possible that the elimination of CaN from a hippocampal pyramidal cell could impact its participation in electrographic seizures. If this were the case, it would confound interpretation of other experimental outcomes. For instance, our goal is to determine whether CaN plays a role in seizure-induced alterations in dendrite anatomy. If elimination of CaN activity suppressed seizure activity, then any anatomic changes could simply be due to the absence of seizures and may not reflect a role for CaN in seizure-induced dendrite development. To address whether a virally transduced neuron participates in bicuculline induced (50 µM) electrographic seizure activity, we combined simultaneous patch clamp and local field potential recordings with multiphoton imaging to correlate spontaneous epileptiform spiking of the CA1 population with action potentials from a virally-transduced pyramidal cell. To accomplish this, we first identified a transfected pyramidal cell by the presence of mCherry in its nucleus and then performed cell-attached patch clamp recordings from that cell while using a second recording electrode to monitor the local field potential. Results from this analysis are shown in Figure 5. Figure 5A is a multiphoton image obtained during a recording session. Several GFP-positive neurons are seen and two are transfected. The recordings in Figure 5B were obtained from the center-most pyramidal cell (see electrode) and show that the activity of this cell is highly correlated with local field potential activity. We analyzed similar recordings from four separate experiments. To quantify the relationship between the activity of the single cells and the CA1 population, we computed the cross-correlation between population spikes in the local field potential recordings and action potentials in the pyramidal cells. The cross-correlation (Fig. 5*C*) revealed a large peak centered at the time of population spike generation (0 ms, see yellow dashed line) which was well above the correlogram for the same activity when time stamps for cell attached spikes were randomly assigned a time during the recordings session (overlaid in red). Based on these findings, we can conclude that individual pyramidal cells, which have been virally transduced and lack CaN activity, fully participate in electrographic seizure activity.

### Knock-out of CaNB1 prevents electrographic seizure-induced reductions in dendrite length and branching

**Figure 6.**
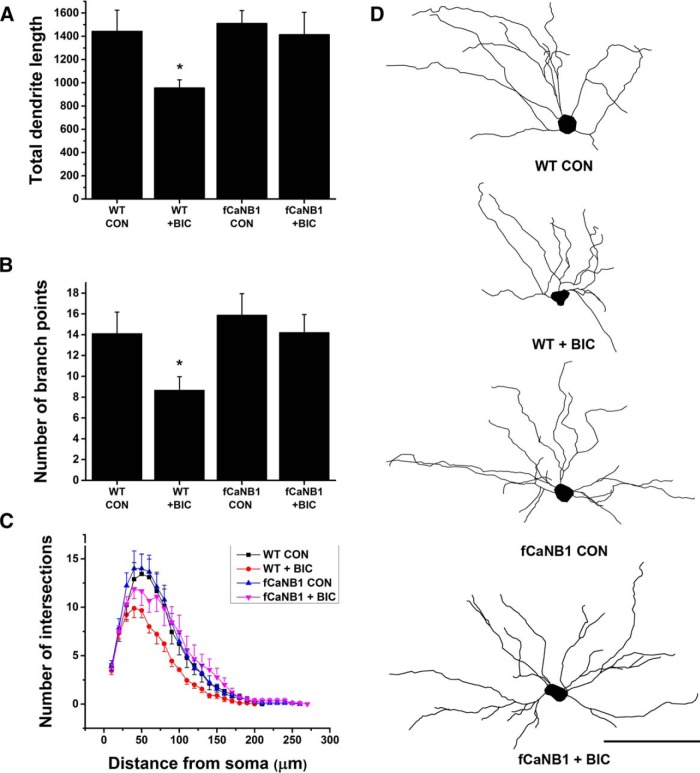
Elimination of CaNB1 from CA1 hippocampal pyramidal cells prevents electrographic seizure-induced reductions of basilar dendrite length and branch number. ***A***, ***B***, Treatment of slice cultures from wild-type (WT) mice for 2 d with bicuculline (BIC 100 µM) results in a decrease in both dendrite length and branch number in mCherry-Cre-transfected neurons. However, an identical treatment of slice cultures from fCaNB1 mice failed to reduce dendrite length or branch number. ***C***, Results of Sholl analyses are consistent with the results in ***A***, ***B***, i.e., dendritic arbors were reduced in length and branching complexity following treatment of transfected neurons from wild-type mice with bicuculline but this effect was greatly attenuated in transfected pyramidal cells from fCaNB1 mice. ***D***, Representative dendrite reconstructions from the four experimental groups. Nine pyramidal cells were reconstructed for each condition. Scale bar, 100 µm; *α = 0.05.

At this juncture, our results indicated that CaNB1 and functional CaN activity was eliminated in viral-transfected neurons and the loss of CaNB1 did not prevent cells from participating in electrographic seizure activity. Given this, we set out to test the hypothesis that CaN plays a role in seizure-induced dendrite growth suppression. To accomplish this, we made slice cultures from 5-d-old wild-type and fCaNB1 littermates and transfected them 1-2 h later with our virus. All mice were homozygous for the Thy1GFP-M transgene, which permitted dendrite reconstructions. On DIV4, half of the slices from wild-type and fCaNB1 mice were treated in a random fashion with bicuculline (100 µM). On DIV6, slices were fixed. Thereafter, the basilar dendrites from GFP-positive CA1 pyramidal cells were reconstructed using Neurolucida. There were four groups of cultures produced by treating slices with vehicle (i.e. control slices) or bicuculline from wild-type and fCaNB1 littermates. For each treatment group, dendrites from transfected neurons with robust mCherry nuclear fluorescence were reconstructed. In keeping with our earlier *in vitro* and *in vivo* work (Nishimura et al., 2008; Nishimura et al., 2011), seizure-like activity in slices from wild-type mice resulted in a significant decrease in both dendrite length and number of dendritic branches compared with wild-type controls (Fig. 6*A*,*B*, length; *p =* 0.026, *t*_(16)_ = 2.34 and branch number; *p =* 0.042, *t*_(16)_ = 2.12). However, in transfected CA1 pyramidal cells from fCaNB1 mice, dendritic length and branch number were unchanged by bicuculline (length fCaNB1: controls 1511.39 ±110.20 µm, bicuculline 1415.47 ± 191.45 µm). Nor were they different from neurons in untreated slices from wild-type animals (length: N.S. *p* = 0.89, *t*_(16)_ = 0.13). Sholl analyses (Fig. 6*C*) further supported the conclusion that the elimination of CaNB1 from CA1 hippocampal pyramidal cells prevented dendrite growth suppression induced by bicuculline treatment. Representative reconstructed dendrites from the four groups of neurons are shown in Figure 6*D*.

## Discussion

In experiments reported here, we employed a novel approach to address the potential molecular mechanisms underlying seizure-induced dendrite growth suppression, which has been hypothesized to contribute to neurobehavioral comorbidities associated with severe childhood epilepsy (Casanova et al., 2014). We focused on the role that the protein phosphatase, CaN, may play in limiting dendrite growth. Using slice culture techniques, we found that the elimination the CaNB1 subunit from CA1 pyramidal cells prevented bicuculline-induced decreases in both the overall length and branching complexity of basilar dendrites. Our electrophysiological recordings showed that these results could not be explained by an inability of transfected neurons to participate in electrographic seizure activity. Immunohistochemical results showed that hSyn-mCherry-Cre viral transfections essentially eliminated CaNB1 from CA1 pyramidal cells taken from floxed fCaNB1 mice. The transfections also prevented the CaN-dependent nuclear translocation of the CREB-regulated transcriptional coactivator CRTC1.

### Early-life seizures induce a CaN-dependent dendrite morphology phenotype

Severe childhood epilepsy is commonly associated with neurobehavioral comorbidities including learning and memory deficits (Camfield and Camfield, 2002; Nolan et al., 2003; Berg, 2011). The clinical term, epileptic encephalopathy, implies that the seizures themselves contribute in important ways to these comorbidities. Indeed, animal experiments using a variety of models have shown that the induction of seizures in early life can impact not only cognition but also socialization, implicating them in intellectual disabilities and autism (Lynch et al., 2000; Lee et al., 2001; Cornejo et al., 2008; Karnam et al., 2009; Nishimura et al., 2011; Talos et al., 2012; Bernard et al., 2013; Lugo et al., 2014). While the behavioral consequences of early-life seizures are now well established in animals, the underlying cellular and molecular mechanisms are under investigation in numerous labs. In 2 different animal models, our group has shown that recurrent seizures during the second and third postnatal weeks not only lead to learning and memory deficits but also dramatic reductions in hippocampal pyramidal cell dendrite length, branching complexity, and spine density (Jiang et al., 1998; Lee et al., 2001; Nishimura et al., 2011), which are reminiscent of pathologic findings in human epilepsy (Swann et al., 2000; Wong, 2005). Epileptiform activity induced in slice cultures has been shown to produce similar effects on dendrite morphology (Nishimura et al., 2008; Casanova et al., 2013). Since these *in vivo* and *in vitro* effects on dendrites are accompanied by decreases in the expression of molecular markers for glutamatergic synapses (Swann et al., 2007a; Swann et al., 2007b), they suggest there is also a decrease in the number of excitatory synapses in the epileptic brain. Since the use-dependent plasticity of glutamatergic synapses is thought to contribute to hippocampal-based learning and memory, it is entirely plausible that the impact of repeated early-life seizures on dendrite microanatomy and synapse number could contribute to the cognitive deficits observed.

Results from clinical investigation and animal studies also suggest that the immature nervous system is uniquely vulnerable to the effects of seizures and that seizures may in some way alter the growth of the brain (Dodrill and Matthews, 1992; Hermann et al., 2008). Indeed, results from our lab have shown that recurrent seizures in infant mice suppress the growth of hippocampal dendrites (Nishimura et al., 2011). Very similar effects have been seen in slice cultures (Nishimura et al., 2008), and results showed that these effects are NMDA receptor dependent implicating downstream calcium signaling mechanisms in growth suppression (Nishimura et al., 2008). Most recently, we were surprised to find that even brief periods (e.g., 4 h) of intermittent seizure-like activity were sufficient to reduced hippocampal pyramidal cell dendrite length and branching complexity (Casanova et al., 2013), and these effects were abolished by the CaN inhibitor, FK506. These results led directly to the studies reported here, where we extended them by knocking out CaNB1 in pyramidal neurons and examining the effects of more prolonged periods of seizure-like activity, when seizures have been shown to block dendrite growth (Nishimura et al., 2008). Our results suggest that CaN plays an important role in the effects seizures have on growing dendrites.

### CaN and seizures: roles in dendritic injury or dendrite development?

Prolonged seizures often lead to neuronal injury and death. Hippocampal neurons are particularly vulnerable in this regard since severe seizures produce a marked loss of hippocampal neurons and gliotic scaring (Cendes et al., 2014). Animals models in which seizures are induced by kainate or pilocarpine in adult rodents are widely used to study the mechanisms and consequences of prolonged seizures. However, recent studies have reported that under certain experimental conditions kainate can induce prolonged seizures without neuronal loss (Zeng et al., 2007). In these instances, the dendrites of neocortical pyramidal cells undergo morphologic changes, which can be followed with time-lapse multiphoton imaging. Results have shown that the dendrites undergo localized swellings which are referred to as “beading.” At the same time, dendritic spines disappear. Once seizures abate, dendrites gradually recover their preseizure morphology in that beading disappears but spine density does not always fully recover. In examining the mechanisms responsible for dendritic injury, investigators have shown that kainite seizures activate cofilin and decrease filamentous actin, which could help explain the morphologic changes in dendrites. Interestingly, the effects on cofilin and dendrite morphology were antagonized by the CaN inhibitor, FK506 (Zeng et al., 2007).

Given these findings, one might suspect that the electrographic seizure-induced morphologic changes in dendrite length and branching reported here could be the result of some form of neuronal injury. Especially since CaN appears to mediate these effects. However, in all of our *in vivo* and *in vitro* experiments, we have never observed signs of neuronal injury including dendritic beading. One possible reason for this is that we have studied the effects of brief seizures *in vivo* and brief intermittent seizure-like activity *in vitro*. Continuous seizure activity has been avoided to prevent neuronal injury. In addition, for many years the developing hippocampus in rodents has been well known to be relatively invulnerable to seizure-induced injury, even after prolonged seizures (Stafstrom, 2002). Thus, it seems unlikely that the elimination of CaN from developing hippocampal neurons in our experiments acts by preventing dendritic injury.

### CaN’s role in normal dendrite development is coopted by seizures

CaN can act over short and extended periods of time to modify neuronal function. During neuronal development, it appears to have an important role in regulating neurite outgrowth. For instance, experiments in the visual system of *Xenopus* have shown that inhibition on CaN activity for 1 or 2 d by the expression of CaN peptide inhibitors increased dendrite arbor complexity (Schwartz et al., 2009). Moreover, experiments in cultured cerebellar granule cells showed that potassium-induced depolarization for one week prevented the later stages of dendrite growth and that this terminal maturation was restored by treatment with the CaN inhibitor, FK506 (Okazawa et al., 2009). Thus, it seems entirely plausible that during dendrite growth CaN normally has a role in limiting the rate of growth. The precise molecular mechanisms underlying these effects of CaN are unknown; however, in *Xenopus*, the effects of the inhibitory peptides were abrogated by the overexpression of NFAT transcription factors, implicating gene transcription in CaN’s ability to suppress dendrite growth (Schwartz et al., 2009). Indeed, CaN could be an important part of a calcium-dependent signaling cascade that tempers growth rates to prevent dendrite overgrowth. In this regard, in Figure 4, we show that the nuclear translocation of the transcriptional coactivator CRTC1 was prevented by eliminating CaN from hippocampal neurons. We also observed a decrease in nuclear CRTC1 in transfected neurons that were not treated with bicuculline (Fig. 4*B*). It is possible that the reduced CRTC1 translocation following seizure-like activity results in part from a shift in CRTC1’s phosphorylation “set point” under baseline conditions. However, we would like to stress that in our studies we used the nuclear translocation of CRTC1 solely to verify the experimental elimination of CaN from hippocampal neurons. Nonetheless, our results could point to CRTC1 as a potential part of a larger signaling cascade regulating dendrite growth.

On the other hand, CaN can act more rapidly in regulate nerve cell growth. For instance, calcium imaging of *Xenopus* spinal cord neurons have demonstrated large spontaneous intracellular calcium waves in growth cones that over a few minutes inhibited neurite extension. These effects were shown not to be dependent on gene transcription (Ribera and Spitzer, 1989) but on CaN actions on actin cytoskeleton (Lautermilch and Spitzer, 2000). In this regard, our previous experiments reported a marked reduction in dendrite arbor complexity after 4 h of electrographic seizure activity and that this effect was abolished by the CaN inhibitor FK506 (Casanova et al., 2013). Thus, CaN may mediate the effects of seizures on developing dendrites over both short and extended periods of time but through different mechanisms. However, taken together our results lead us to suggest that the increases in intracellular calcium that accompany recurring seizure activity leads to hyperactivation of CaN and the suppression of dendrite growth.

## Synthesis

Reviewing Editor: Alfonso Represa, INSERM Decisions are customarily a result of the Reviewing Editor and the peer reviewers coming together and discussing their recommendations until a consensus is reached. When revisions are invited, a fact-based synthesis statement explaining their decision and outlining what is needed to prepare a revision will be listed below. The following reviewer(s) agreed to reveal their identity: Paolo Calabresi.

Dear authors,

I have taken into consideration here the criticisms and rebuttal of the previous version (submitted to J Neurosci) and the comment of a new reviewer that considered the paper of interest and suitable for publication with only a few comments (below), aimed to improve the manuscript, and that should be taken into account for acceptance:

1. Introduction: the rationale of the study should be better explained. This point was somehow criticized before. I think that the main information required for understanding the manuscript is presented there, but maybe that you can better describe the Question, the Objective and the Approach in a conclusive paragraph.

2. Materials and methods: please provide additional methodological details for the electrophysiological experiments (also see below).

3. For the LFP experiments, please provide a scale bar in figure 5 and accurately describe how the LFP is evoked in the Methods section of the manuscript.

4. What is the solution used to fill electrodes for the extracellular recordings?

5. For epileptic like activity the GABAA receptor antagonist bicuculline is used. Although the slice preparation used for the immunohistochemistry and electrophysiology experiments is the same, the concentration of bicuculline chosen for these experiments is different. How the authors can explain this?

6. Hippocampal slice cultures are treated from 15 minutes to 8 hours with bicuculline. What is the criterion used for the choice of the time point in each experiment?

7. Page 20, line 450: the authors should indicate the concentration of bicuculline used.

8. There is no correspondence between the concentration of bicuculline indicated in the main text (100 µM) and in the legends (50 µM).

9. Please revise the text for typo errors.

10. Please separate the Introduction section by the Significance one.

11. Abbreviations used should be consistent thought the text.
